# *In Situ* Dividing and Phagocytosing Retinal Microglia Express Nestin, Vimentin, and NG2 *In Vivo*

**DOI:** 10.1371/journal.pone.0022408

**Published:** 2011-08-05

**Authors:** Stefanie G. Wohl, Christian W. Schmeer, Thomas Friese, Otto W. Witte, Stefan Isenmann

**Affiliations:** 1 Hans Berger Clinic of Neurology, Jena University Hospital, Jena, Germany; 2 Department of Neurology, HELIOS Klinikum Wuppertal, Wuppertal, Germany; 3 University of Witten/Herdecke, Witten, Germany; Universidade Federal do Rio de Janeiro, Brazil

## Abstract

**Background:**

Following injury, microglia become activated with subsets expressing nestin as well as other neural markers. Moreover, cerebral microglia can give rise to neurons *in vitro*. In a previous study, we analysed the proliferation potential and nestin re-expression of retinal macroglial cells such as astrocytes and Müller cells after optic nerve (ON) lesion. However, we were unable to identify the majority of proliferative nestin^+^ cells. Thus, the present study evaluates expression of nestin and other neural markers in quiescent and proliferating microglia in naïve retina and following ON transection in adult rats *in vivo*.

**Methodology/Principal Findings:**

For analysis of cell proliferation and cells fates, rats received BrdU injections. Microglia in retinal sections or isolated cells were characterized using immunofluorescence labeling with markers for microglia (e.g., Iba1, CD11b), cell proliferation, and neural cells (e.g., nestin, vimentin, NG2, GFAP, Doublecortin etc.). Cellular analyses were performed using confocal laser scanning microscopy. In the naïve adult rat retina, about 60% of resting ramified microglia expressed nestin. After ON transection, numbers of nestin^+^ microglia peaked to a maximum at 7 days, primarily due to *in situ* cell proliferation of exclusively nestin^+^ microglia. After 8 weeks, microglia numbers re-attained control levels, but 20% were still BrdU^+^ and nestin^+^, although no further local cell proliferation occurred. In addition, nestin^+^ microglia co-expressed vimentin and NG2, but not GFAP or neuronal markers. Fourteen days after injury and following retrograde labeling of retinal ganglion cells (RGCs) with Fluorogold (FG), nestin^+^NG2^+^ microglia were positive for the dye indicating an active involvement of a proliferating cell population in phagocytosing apoptotic retinal neurons.

**Conclusions/Significance:**

The current study provides evidence that in adult rat retina, a specific resident population of microglia expresses proteins of immature neural cells that are involved in injury-induced cell proliferation and phagocytosis while transdifferentiation was not observed.

## Introduction

Microglia constitute immune competent cells of the central nervous system (CNS) including the neural retina [1], [2]. In naïve tissue, the cells continuously survey their microenvironment via extremely motile processes [3]. Microglia are involved in the inflammatory response after injury as well as in major neurodegenerative diseases of the CNS. Injury-induced neuronal cell death in the brain and retina leads to activation of microglial cells [4], [5]. Depending on the lesion type, they change their morphology from ramified into ameboid, proliferate, secrete cytokines to induce cell proliferation, e.g. of macroglia, secrete chemokines to attract other immune cells, and accumulate at the lesion site [5], [6]. In particular, transection of the ON, and, therefore, of projecting axons from (RGCs), leads to delayed apoptotic cell death within 4–5 days after injury with a peak at day 7 [7], [8], [9]. Within this time, resident retinal microglia proliferate *in situ* [10] and phagocytose debris from dying RGCs [11], [12]. The blood-retinal barrier (BRB) is not affected following an ON lesion, and there is no increased cell infiltration of hematogenously-derived inflammatory cells [13], [14], [15]. Thus, an ON lesion is an appropriate model for analyzing intrinsic immunological and cellular response mechanisms.

After injury in the brain or spinal cord of adult rats, subsets of activated microglia have been reported to transiently express markers of immature neural cells including nestin [16], [17] and the chondroitin sulfate proteoglycan NG2 [18], [19], [20], [21], which was primarily described for oligodendrocyte precursor cells [22], [23]. Moreover, *in vitro* studies suggest that nestin and NG2 expression in cerebral microglia is an indication of a rather immature phenotype with high plasticity similar to that found in the neonate brain [21], [24].

In a previous study, we evaluated cell proliferative responses and nestin re-expression from cells with known neurogenic potential in the retina, i.e. Müller cells and astrocytes following an ON lesion [25]. Both cell populations expressed nestin, albeit at a low proliferation rate. Moreover, the majority of dividing cells in the injured retina were identified as resident microglia. Interestingly, the transient increase in microglial cell number was due to local cell division [10]. Nestin expression was not restricted to activated macroglial and blood vessel cells, i.e. endothelial cells and pericytes, as already described [26], [27], [28], but this intermediate filament was also present in another cell type identified herein as resident parenchymal retinal microglia.

Recently, nestin^+^ microglia were also observed in the naïve brain. Their numbers were dependent on the cerebral region analysed [29]. Nestin is thought to be responsible for changes in the cytoskeleton and, consequently, the cell shape [29]. In addition, nestin expression is associated with migration and proliferation of immature cells [30], [31], particularly the neural progenitor cells (NPCs) [32], [33], as well as non-neural cell types [30], [31]. To our knowledge, there are no reports in the literature regarding expression of nestin on adult retinal microglial cells. Furthermore, the role of this “ectopic” nestin expression only in subpopulations of microglia in the adult central nervous system (CNS), especially after injury, has not been completely clarified.

The purpose of the present study was to evaluate the expression of nestin and other “ectopic” neural proteins, including markers of immature and mature glial and neuronal cells, in resting resident and activated retinal microglia after a distal ON injury. We further addressed the question of whether nestin expression by microglial cells is associated with cell division and phagocytosis as well as possible transdifferentiation processes.

## Materials and Methods

### Animals

Adult female Sprague Dawley rats (230–280 g) obtained from Charles River Laboratories, Sulzfeld, Germany were maintained in standard cages under a 12 h light/12 h dark cycle with free access to food and drinking water. Rats were kept in accordance with the European Convention for Animal Care and Use of Laboratory Animals. All experiments were approved by the local Animal Care Committee (Thueringer Landesamt, Weimar, Germany, permit number 02-11/04).

### ON transection

**Figure 1:**
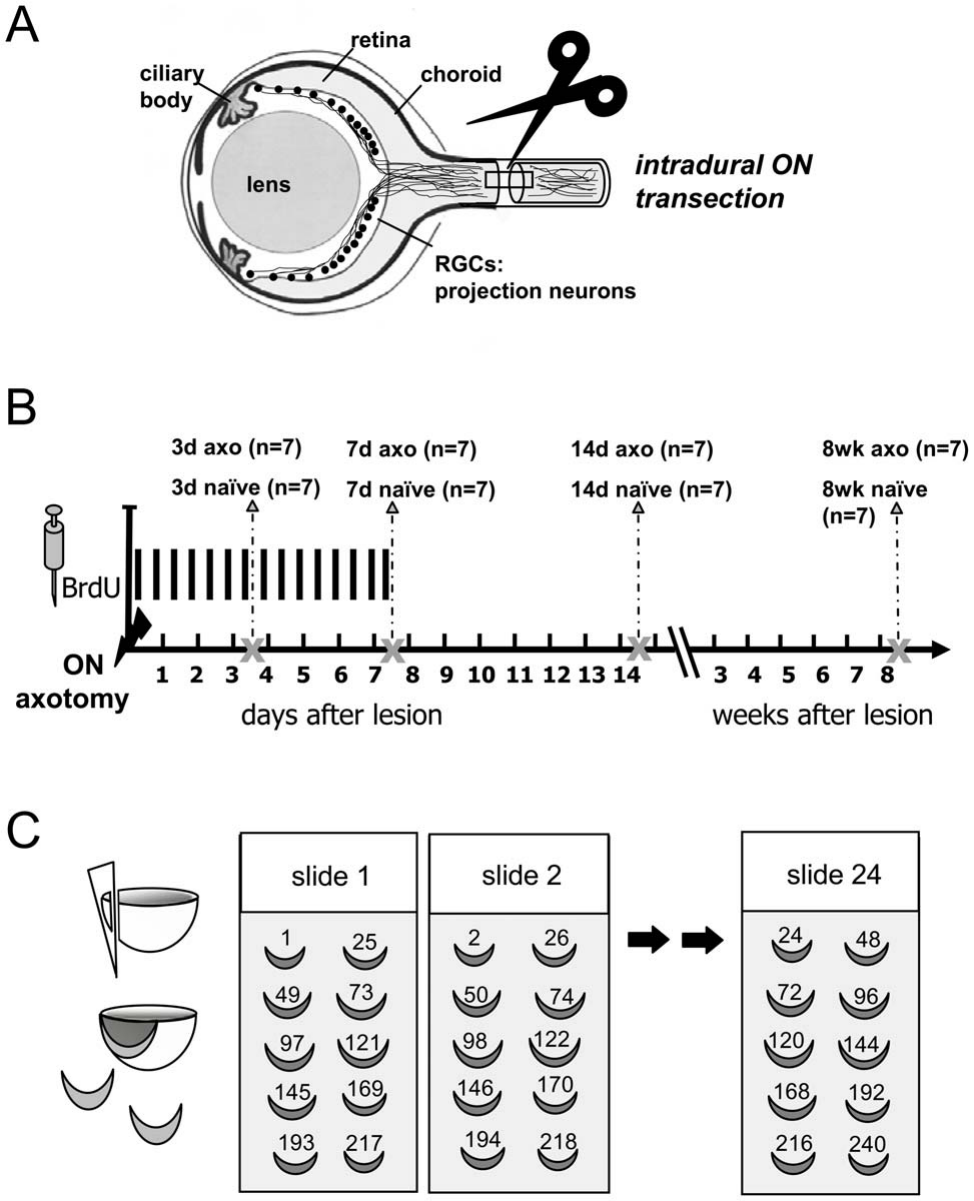
Experimental design. A: preparation of distal optic nerve (ON) lesion; retinal ganglion cell (RGC) axons were intradurally transected approx. 2 mm behind the eyeball. B: immediately after surgery, rats received intraperitoneal injections of BrdU twice daily up to 3 or 7 days. Animals were sacrificed at days 3, 7, and 14 or 8 weeks after ON axotomy (indicated by an X in the time axis; for details, see Materials and Methods). Cell fate analyses were performed 14 days and 8 weeks after injury. Each group consisted of 7 rats. C: Every 25^th^ horizontal cross section of the eye cup including the neural retina was placed on consecutive slides (24 slides in sum, 10 sections per slide) resulting in a representative coverage of the whole retina on one slide.

Surgery on the animals was performed as described in detail elsewhere [34]. Briefly, following anaesthesia by means of an i.p. injection of chloral hydrate (7% in PBS, 420 mg/kg Sigma-Aldrich, Taufkirchen, Germany), skin and connective tissue were incised, and the optic nerve was exposed and transected intradurally approximately 2 mm distal to the eye bulb (Fig. 1A). For retrograde RGC labeling, a small piece of gel foam soaked in a 5% aqueous solution of the fluorescent dye Fluorogold (FG; Fluorochrome Inc., Denver, CO, USA) was placed on the ON stump immediately after axotomy (axo) [34]. Since apoptotic RGCs are phagocytosed by microglia, the cells are also selectively labeled with the dye [35], [36]. Unoperated animals were used as controls.

### Bromodeoxyuridine (BrdU) administration

Both lesion and control rats were anesthetized by being subjected to inhaling 2.0% isoflurane in an oxygen/nitrous oxide (1∶2) mixture. Thereafter, 5,2-bromodeoxyuridine (BrdU, 50 mg/kg, dissolved in sterile saline, Sigma-Aldrich, Taufkirchen, Germany) was injected i.p. as previously described [25]. BrdU was given twice daily starting after surgery for up to 3 or 7 days (Fig. 1B).

### Tissue preparation

A total of seven animals for each time period were sacrificed by an overdose of chloral hydrate at days 3, 7 and 14 days, or 8 weeks after ON transection. *For immunostaining*, eyes were enucleated, fixed by immersion for 20 min in 4% paraformaldehyde (PFA), and eye cups incubated overnight in 30% sucrose (solution in phosphate buffered saline [PBS]; Sigma, Germany). Eye cups, including the neural retina, frozen in embedding medium (Tissue Tek, Sakura, Germany) were cryosectioned into 240 sections (25 µm thickness). Every 25^th^ section was placed on consecutive slides (24 slides in sum, 10 sections per slide) to attain a representative coverage of the retina on one slide (Fig. 1C). All slides were air-dried.

*For immunopanning*, eyes were enucleated, retinae were explanted and prepared in Hank's basal salt solution (HBSS, Sigma-Aldrich, Taufkirchen, Germany) containing 25 mL HBSS, 3 mg/mL bovine serum albumin (BSA) and 15 mM HEPES (4-[2-hydroxyethyl]-1-piperazineethanesulfonic acid, Invitrogen, Darmstadt, Germany). Tissue was enzymatically dissociated using papain (18 U/mL, Sigma-Aldrich, Germany) for 20 min at 37°C. Ovomucoid solution (Sigma-Aldrich, Germany) was added and the tissue mechanically dissociated. Dissociated cells were centrifuged (8 min, 350×g), resuspended in PBS and transferred to pre-coated Petri dishes, as previously described [37]. Briefly, two 30 mm Petri dishes were incubated with affinity-purified horseradish peroxidase (HRP) coupled goat anti-mouse IgG (10 mg/ml, Dianova, Germany) overnight at 4°C. Primary antibody mouse anti-rat-CD11b (1∶20; with 0.2% BSA in PBS) was added and dishes incubated for 1.5 h at room temperature (RT). Cell suspensions were added to Petri dishes and incubated for 30 min at RT. Dishes were gently washed with PBS and cells fixed with 2% PFA.

### Immunofluorescence

To identify retinal microglia, antibodies against the following three different proteins were used: calcium binding protein Iba1 [38], [39], surface receptor protein CD11b (OX-42, clone MCR) [40], and the glycoprotein macrosialin (the murine equivalent of human CD68, clone ED1) [2], [41]. Since CD68 only labels a minor fraction of retinal microglia, this marker was not appropriate for our purposes. We also used *Lycopersicon esculentum* (tomato) Lectin that binds N-acetylglucosamine oligomers and is an effective marker of microglial cells in rodents [39], [42]. Phagocytising microglia were identified using an antibody against TREM2, a receptor responsible for recognising, binding and uptake of apoptotic cells [43], [44]. Microglial nestin expression was analysed using the monoclonal mouse anti-rat antibody clone 401 [45], [46] that has been reported in a variety of studies regarding neurogenesis in brain, and also in studies of cerebral microglia [24], [29]. For vimentin and NG2 labeling, we used antibodies already described elsewhere for microglial assays in CNS [18], [21], [29], [47], [48].

#### Retinal sections

retinal slices were fixed with 4% PFA. BrdU and Ki67 staining was performed as previously described using 2 N HCl for 20 min at 37°C, followed by incubation with 0.1 M borate buffer (pH 8.5) for 10 min at RT, and/or heat induced antigen retrieval (HIAR) with EDTA buffer at pH 8.0. For tissue labeling, a standard staining protocol was used as previously described [10]. Briefly, retinal slices were incubated with primary antibodies dissolved in 2% normal donkey serum (NDS) solution overnight at 4°C. Antibodies used for the various combinations of double and triple staining are shown in Table 1. Washing was followed by incubation with secondary antibodies in 10% NDS solution for 1 h at RT. Secondary antibodies constituted Rhodamine conjugated donkey anti-rat IgG, Rhodamine conjugated donkey anti-rabbit IgG, Rhodamine conjugated donkey anti mouse IgG (each 1∶1000, Dianova, Germany), Cy5 conjugated donkey anti-mouse IgG, Cy5 conjugated donkey anti-goat IgG, Cy5 conjugated donkey anti-rabbit IgG (each 1∶500 Dianova, Germany), Alexa Fluor 488 conjugated donkey anti-goat, Alexa Fluor 488 conjugated donkey anti-mouse, and Alexa Fluor 488 conjugated donkey anti-rabbit (each 1∶250, Molecular Probes, Germany). When two primary antibodies from the same species were used, incubation with Fab-fragments (Rhodamine conjugated donkey anti-mouse or -donkey anti-rabbit, each 1∶50, Dianova, Germany) was undertaken. Cell nuclei were counter-stained with DAPI (4,6-diamino-2-phenylindole). For the time course analysis of apoptotic neural cell death and confirmation of vital BrdU labeling, terminal deoxynucleotidyl transferase-mediated dUTP nick-end labeling (TUNEL) was performed using a cell-detection kit (Fluorescein In Situ Cell Detection Kit; Roche Applied Science, Germany) as described previously [10], [49].

**Table 1:** Primary antibodies employed.

MARKER (SPECIES, IgG TYPE)	DETECTION OF/CELLULAR PHENOTYPE	DILUTION	DISTRIBUTOR/SOURCE (CATALOG NUMBER)
**Iba1** rabbit IgG	microglia, macrophages	1∶500	Wako, Neuss, Germany, (019-19741)
**CD11b** mouse IgG2a	microglia, macrophages	1∶100	AbD Serotec, Düsseldorf, Germany (MCA275R)
**CD68** mouse IgG1	microglia, macrophages	1∶100	AbD Serotec, Düsseldorf, Germany (MCA341R)
**mTREM2** sheep IgG	phagocytosing microglia/macrophages	1∶100	R&D Systems, Minneapolis, USA (AF-1729)
**nestin** mouse IgG	astrocytes, Müller glia, NSC/PCs, microglia	1∶100	BD Bioscience, Heidelberg, Germany (5563909)
**vimentin** mouse IgG or goat IgG	astrocytes, Müller glia, NSC/PCs, microglia	1∶100	Sigma-Aldrich, Taufkirchen, Germany (V6389); Santa Cruz, Heidelberg, Germany (sc-7557)
**GFAP** mouse IgG or rabbit IgG	astrocytes, Müller glia	1∶750/1∶500	Millipore, Germany (MAP360) DAKO, Glostrup, Denmark (Z0334)
**NG2** rabbit IgG	OPCs, NG2 glia, microglia	1∶100	Millipore, Germany (AB5320)
**BrdU** rat IgG2a	proliferating cells, S-phase of cell cycle	1∶250	AbD Serotec, Düsseldorf, Germany (OBT0030CX)
**Ki67** rabbit IgG 7	proliferating cells, all phases of cell cycle	1∶100	Novocastra, Newcastle, UK (NCL-Ki67p)
**NeuN** mouse IgG	neurons	1∶200	Millipore, Germany (MAB377)
**Doublecortin (Dcx)** goat IgG	neuronal precursor cells	1∶250	Santa Cruz, Heidelberg, Germany (sc-8066)
**β III Tubulin (TUJ1)** mouse IgG2a	neurons	1∶500	Covance (Hiss) Freiburg, Germany (MMS-435P)
**Brn3a** goat IgG	retinal ganglion cells	1∶250	Santa Cruz, Heidelberg, Germany (sc-31984)
**glutamine synthetase** mouse IgG	Müller glia, astrocytes	1∶250	Millipore, Germany (MAB 302)
**von Willebrandt factor** rabbit IgG	endothelial cells of blood vessels	1∶100	Dako, Glostrup, Denmark (IR527)
Fluorescein labeled *Lycophyllum* **(tomato) lectin**	microglia, endothelial cells of blood vessels	1∶100	Vector labs, Burlingame, USA (FL-1171)

#### Isolated microglia

Primary antibodies (Table 1) were dissolved in 5% NDS-solution supplemented with 3% BSA in PBS, 0.2% Triton X-100, and incubated for 1 h at RT. After washing with PBS for 10 min, the dishes were incubated for 30 min at RT with the secondary antibodies (see above). Subsequently, DAPI was added for 5 min. Dishes were then washed for 10 min in PBS and embedded with Moviol (Calbiochem, Germany).

To determine the specificity of primary antibody-binding, sections and isolated cells were incubated only with secondary antibodies.

### 3-dimensional cell analyses

Microglial marker co-localization in sections as well as after cell isolation was exclusively and extensively analysed via z-dimension stacked micrographs using a confocal laser-scanning microscope (LSM 510 Meta and 710 Meta, Zeiss, Jena, Germany). The “Ortho-”, “Gallery-” or “3D-function” from ZEN software (Zeiss, Germany) was employed for cell analysis. To illustrate 3-dimensionality and to show whole cellular structures, especially with regard to branched microglial processes that are difficult to ascertain in a single optical slice, the presented figures are mainly shown as merged images of all optical slices of a cell z-stack.

### Cell counts

Total numbers of Iba1^+^, BrdU^+^, and Ki67^+^ cells were assessed for every 5^th^ retinal section. The numbers of microglia co-localizing for nestin, BrdU, and Ki67 were determined for every 10th section. For all other markers, co-expression was analyzed for every 25^th^ section. Analysis of differential expression from specific markers over time was used to identify several microglial phenotypes in naïve and lesioned retinas. Absolute numbers of particular microglial phenotypes were evaluated per section, as already described for retinal studies [50] and results were related to previous studies [10], [25]. To confirm *in vivo* observations and to exclude the possibility that adjacent structures, e.g. macroglial processes lead to false interpretations, co-localization and numbers of nestin^+^ microglia were also estimated after immunopanning. Four naïve or lesioned retinae were pooled for every approach that was repeated for every condition. Ten 400 µm×400 µm areas in the Petri dish were precisely scanned and numbers of microglia as well as numbers of nestin^+^ microglia were evaluated. Relative numbers of stained cells are given as percentage of the total cell count. All values are given as mean ± standard error of the mean (S.E.M.). Since BrdU labeling is cumulative, corresponding controls for every time point were evaluated. Total numbers of microglia as well as the nestin^+^ fraction were not different over time, and therefore control values were averaged.

### Statistical analysis

Each group consisted of at least 7 animals. Significant differences between the means from lesion and corresponding control groups and between the different time points after lesion were assessed using the Mann-Whitney test (U-test, p<0.05). Differences between cell fractions within a group were determined using the Wilcoxon test (p<0.05). In addition, the α adjustment required for multiple testing was performed using the Holm-Bonferroni Method.

## Results

### Resting retinal microglia express nestin

**Figure 2:**
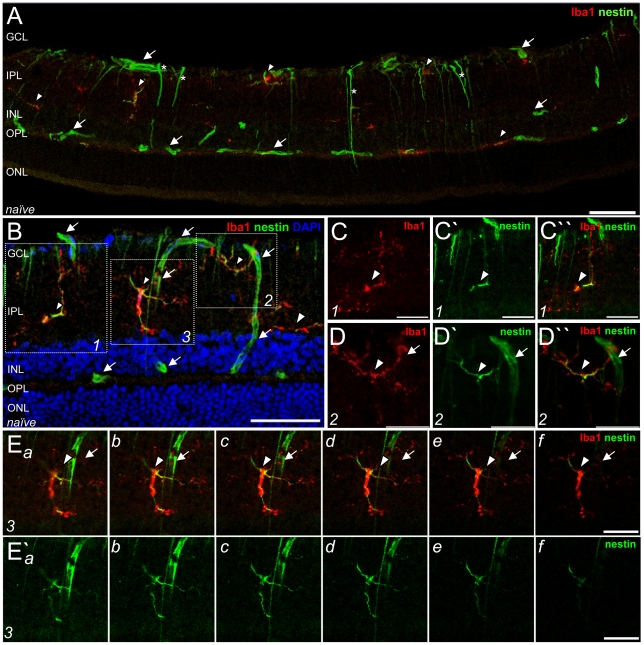
Nestin^+^ microglia in the naïve retina. Immunofluorescent labeling with Iba1 (red) and nestin antisera (green) as well as DAPI nuclear staining (blue). Resting microglia, mainly found in the GCL and IPL, had fine branched processes which expressed nestin in some, but not all processes (A,B arrowheads; boxes 1 to 3 in B, higher magnification in C-C″, D-D″ and E-E′, respectively). Ea–f and E′a–f represent the gallery of 1 µm optical sections of this z-stack. Nestin was also found in retinal blood vessels (arrows) and in few radial macroglial processes (asterisks). The micrographs in A–D are merged z-stacked images of 1 µm optical sections to illustrate the entire cell dimension. GCL: ganglion cell layer, IPL: inner plexiform layer, INL: inner nuclear layer, OPL: outer plexiform layer, ONL: outer nuclear layer. Scale bar (A,B) 50 µm, (C-C″,D-D″, E,E′) 20 µm.

In the adult rat retina, considerable numbers of ramified Iba1^+^ microglia were located in the plexiform layers and the ganglion cell layer (GCL) of the neural retina (Fig. 2A,B arrowheads). All retinal microglia were also positive for CD11b and tomato lectin. In the naïve retina, nestin was sparsely expressed (Figs. 2A,B). Interestingly, short, horizontally oriented nestin filaments were observed in processes belonging to most resting microglia, especially in the retinal plexiform layers (Fig. 2B boxes 1 and 2, higher magnification in C-C″,D-D″, box 3 is shown as a gallery view of the z-stack in E,E′a–f arrowheads, Video S1). Nestin filaments were also observed in blood vessels (Figs. 2A,E, arrows) and in few processes of retinal astrocytes and Müller glia spanning radially through the retinal layers (Fig. 2A, asterisks). Under physiological conditions, about 150 microglia/section were found in the retina proper and, of these, approximately 60% expressed nestin.

### Retinal microglia activation and nestin up-regulation after ON axotomy

**Figure 3:**
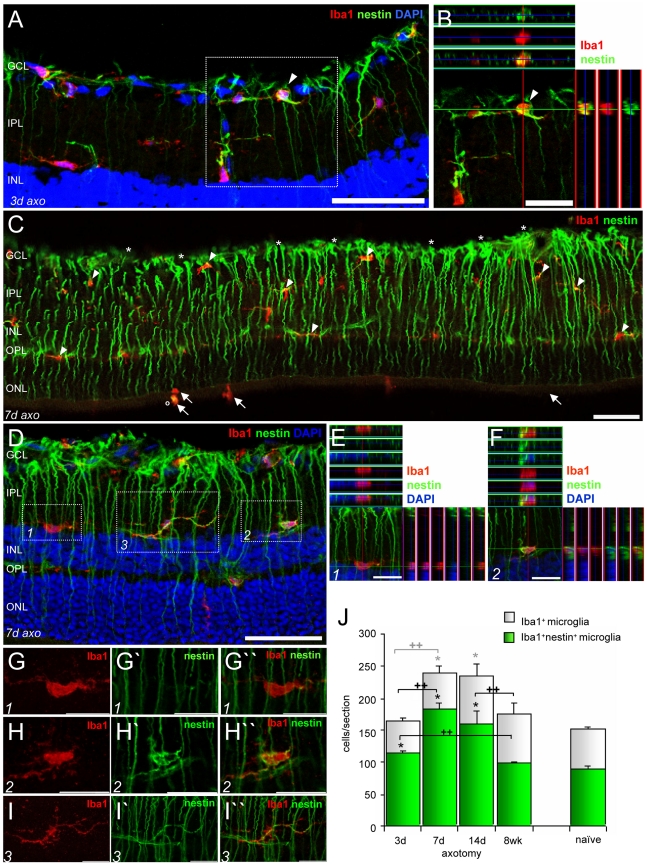
Retinal nestin^+^ microglia after ON axotomy. A–I: Immunofluorescent labeling with Iba1 (red), nestin (green) and DAPI (blue). 3 days after ON axotomy, rather rounded nestin^+^ microglia were found in the GCL and IPL (A, arrowhead-marked cell in B). C–I: 7 days after ON lesion, a significant increase in nestin immunoreactivity was observed predominantly in the processes of astrocytes and Müller glia (C, examples are illustrated with asterisks). Increased numbers of Iba1^+^ microglia were mainly found in the inner retinal layers and the OPL, where the majority express nestin (arrowheads). A few ameboid Iba1^+^ cells observed in the photoreceptor layer (arrows) were also nestin^+^ (o). Some retinal microglia lacked nestin filaments (D, box 1, as ortho view in E, higher magnification in G-G″), however, the majority expressed nestin either perinuclearly (D, box 2, as ortho view in F, higher magnification in H-H″) or in their long processes (D, box 3, higher magnification in I-I″). J: Absolute numbers of total and nestin^+^ microglia 3, 7, 14 days, and 8 weeks after ON transection and in the naïve retina. 7 to 14 days after ON axotomy, the number of Iba1^+^ microglia was significantly increased compared to naïve controls, while the number of nestin^+^ microglia was already significantly increased 3 days after injury, reaching a maximum on day 7. Mean ± S.E.M., significant differences between lesion and corresponding control groups (* p<0.05, ** p<0.01), and between the lesion or control groups over time (+ p<0.05, ++ p<0.01) are indicated: grey and black symbols are used for the white and green diagrams, respectively. The micrographs in A,C,D,G–I are merged z-stacked images of 1 µm optical sections. GCL: ganglion cell layer, IPL: inner plexiform layer, INL: inner nuclear layer, OPL: outer plexiform layer, ONL: outer nuclear layer, axo: axotomy. Scale bar (A,C,D) 50 µm, (B,E,F,G-G″,H-H″,I-I″) 20 µm.

Three days after ON transection, a fraction of retinal microglia underwent a morphological change to become hypertrophic though there was no marked increase in numbers (Figs. 3A,J). Some of the activated microglia were found in the GCL adjacent to the lesioned RGCs and expressed nestin in the soma (Fig. 3A, the arrowhead-marked cell in the box is shown in B). After 7 days, there was an apparent increase in nestin immunoreactivity in the retinal astrocytes in the GCL, and in radial Müller glia (Fig. 3C, asterisks) indicating an injury-induced macroglial response. At this time point, the number of retinal microglia and also those expressing nestin were significantly increased as compared to controls and also to the 3-day post injury group (Figs. 3C, arrowheads; 3J). Although nestin was not expressed in every single microglial cell (Fig. 3D box 1 is shown in E as ortho view, and higher magnification in G-G″), nestin^+^ retinal microglia displayed either a rather ameboid (Fig. 3D box 2, shown in F, and higher magnification in H-H″) or a highly ramified morphology (Fig. 3D box 3, higher magnification in I-I″, Video S2). In ameboid microglia, nestin was predominantly found around the nucleus and in some truncated processes (Figs. 3E, higher magnification in H-H″). In ramified microglia, nestin filaments were mainly located in the processes (Fig. 3D box 3, higher magnification in I-I″). Quantification of absolute numbers of microglia (Fig. 3J) revealed a maximum 60% increase in the number of retinal microglia 7 to 14 days after ON transection. After 8 weeks, the number of microglia declined to baseline levels. However, the number of nestin^+^ microglia had significantly increased as early as 3 days post ON transection compared to naïve tissue. Maximum numbers were reached between 7 and 14 days, representing an increase of 14% (up to 74%) compared to those obtained after 3 days. After 8 weeks, the number of nestin^+^ microglia declined to control levels.

**Figure 4:**
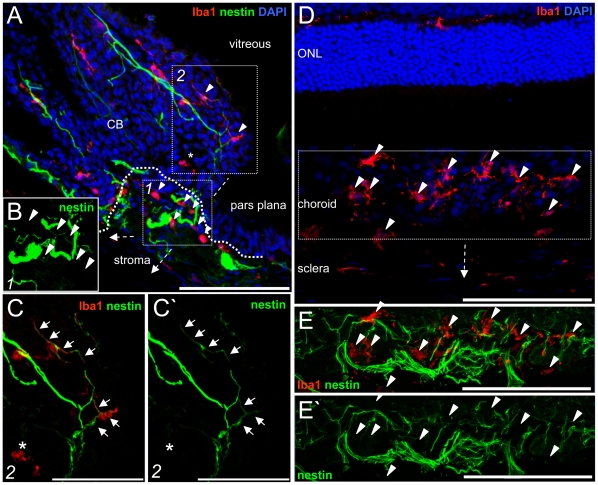
Nestin expression in the ciliary body and choroid. Immunofluorescent labeling with Iba1 (red), nestin (green), and DAPI (blue). A–C: In the ciliary stroma, the area below the dotted white line, Iba1^+^nestin^−^ macrophages were found (A, box 1, higher magnification in B, arrowheads). However, in the ciliary epithelium, branched Iba1^+^ cells had nestin filaments in some of their processes (A, box 2, higher magnification in C,C′ arrows). Ameboid cells in the epithelium were nestin^−^ (C,C′ asterisk). In the choroid, many ameboid Iba1^+^ macrophages (D,E arrowheads) were found within numerous nestin filament bundles (E,E′), however these cells *per se* were nestin^−^. The micrographs in the figures are merged z-stacked images of 1 µm optical sections to illustrate the entire cell dimension. ONL: outer nuclear layer, CB: ciliary body. Scale bar in (A,D-E′) 100 µm, (C-C′) 50 µm.

In addition, ameboid Iba1^+^ cells in the photoreceptor layer (Fig. 3C, arrows) and ramified Iba1^+^ cells in the ciliary epithelium (Fig. 4A) also expressed nestin (Figs. 3C, circle; 4A box 2, higher magnification in C,C′ arrows). Large Iba1^+^ macrophages in the ciliary stroma (Fig. 4A box 1, higher magnification in B) and choroid (Figs. 4D,E,E′, arrows) were embedded in nestin filament bundles, but these cells *per se* were nestin^−^ in both naïve and lesioned tissue.

### Numbers of nestin^+^BrdU^+^microglia increase after ON transection

**Figure 5:**
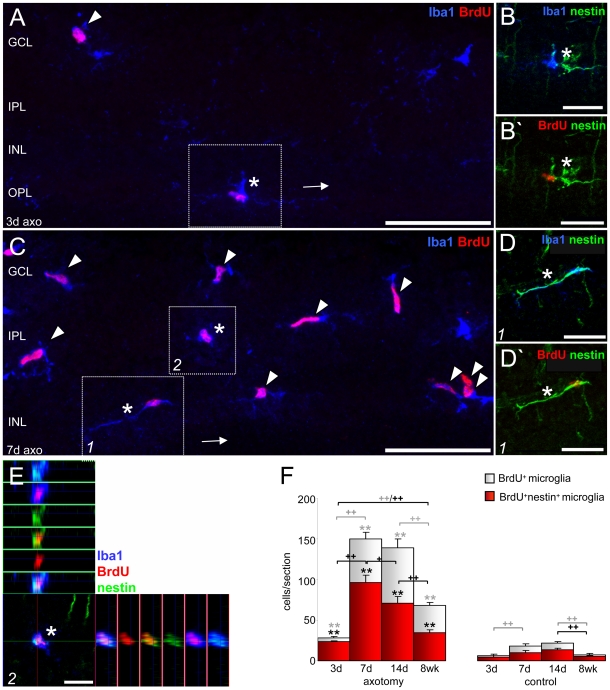
Numbers of retinal BrdU^+^ microglia increase after ON axotomy. A–E: Immunofluorescent labeling with Iba1 (blue), BrdU (red), and nestin antisera (green). Three days after ON axotomy, some microglia observed in inner retinal layers were BrdU^+^ (A, arrowhead) and some also nestin^+^ (the asterisk-marked cell in the box is shown in B,B′). After 7 days, BrdU^+^Iba1^+^ microglia (C, arrowheads) increased in number, especially in the GCL/IPL, and most of them co-expressed nestin (asterisk-marked cell in box 1 is shown D,D′; that of box 2 as ortho view in E). F: Absolute numbers of BrdU^+^ and nestin^+^BrdU^+^ microglia 3, 7, 14 days, and 8 weeks after ON transection and in the naïve retina. At all time points analyzed, numbers of BrdU^+^ and nestin^+^BrdU^+^ microglia were increased after ON axotomy compared to corresponding controls, reaching maximum numbers 7–14 days after injury. Mean ± S.E.M., significant differences between lesion and corresponding control groups (* p<0.05, ** p<0.01), and between the lesion or control groups over time (+ p<0.05, ++ p<0.01) are indicated: grey and black symbols are used for the white and red diagrams, respectively. The micrographs in A-D′ are merged z-stacked images of 1 µm optical sections to illustrate the entire cell dimension. GCL: ganglion cell layer, IPL: inner plexiform layer, INL: inner nuclear layer, OPL: outer nuclear layer, axo: axotomy. Scale bar (A,C) 50 µm, (B,B′,D,D′) 20 µm, (E) 10 µm.

Proliferation potential of resident microglia over time was analysed using BrdU labeling (Fig. 5). As early as 3 days after ON axotomy, numbers of BrdU^+^ microglia were significantly increased compared to the corresponding control (Figs. 5A,F). However, 7 days after ON lesion, the quantity of BrdU^+^ microglia showed a five-fold increase than on day 3 and, moreover, remained high up to day 14 (Figs. 5C,F). Microglia that proliferated in response to injury were defined here as activated microglia. In unlesioned controls, an increase in proliferating microglia due to continued BrdU injections up to day 7 (8 further injections, see Fig. 1) was observed from days 3 to 7, and there was a decrease in cell numbers from day 14 up to 8 weeks as a probable consequence of microglial death over time.

The numbers of BrdU^+^nestin^+^ microglia 3 days after ON injury, (Fig. 5A, the asterisk-marked cell in the box is shown in B,B′) were four times higher than in naïve tissue representing about 80% of all BrdU^+^ microglia. Thus, approximately 20% of all BrdU^+^ microglia were nestin^−^ (Fig. 5F). Seven days after ON axotomy, the number of BrdU^+^nestin^+^ microglia (Fig. 5C, box 1 is shown in D-D′, box 2 is displayed as ortho view in E) reached a maximum and, moreover, over half of all nestin^+^ microglia were now BrdU^+^ (Fig. 5F). After 14 days, BrdU^+^ nestin^+^ microglia significantly decreased compared to the numbers at day 7 and further decreased over time. However, after 8 weeks, BrdU^+^nestin^+^ microglia were still significantly increased in comparison to corresponding controls and also significantly increased compared to 3 days post ON lesion, indicating that most of the newly generated cells persisted for several weeks and retained their nestin filaments. On the other hand, the fraction of BrdU^+^nestin^−^ microglia also increased over time, suggesting that two different microglia populations may proliferate in the acute phase after ON axotomy, namely nestin^+^ and nestin^−^ cells. Moreover, it appears that nestin^+^ microglia represent the population with an early response, while nestin^−^ microglia show a delayed response.

### Nestin^+^ microglia proliferate in situ after ON transection

**Figure 6:**
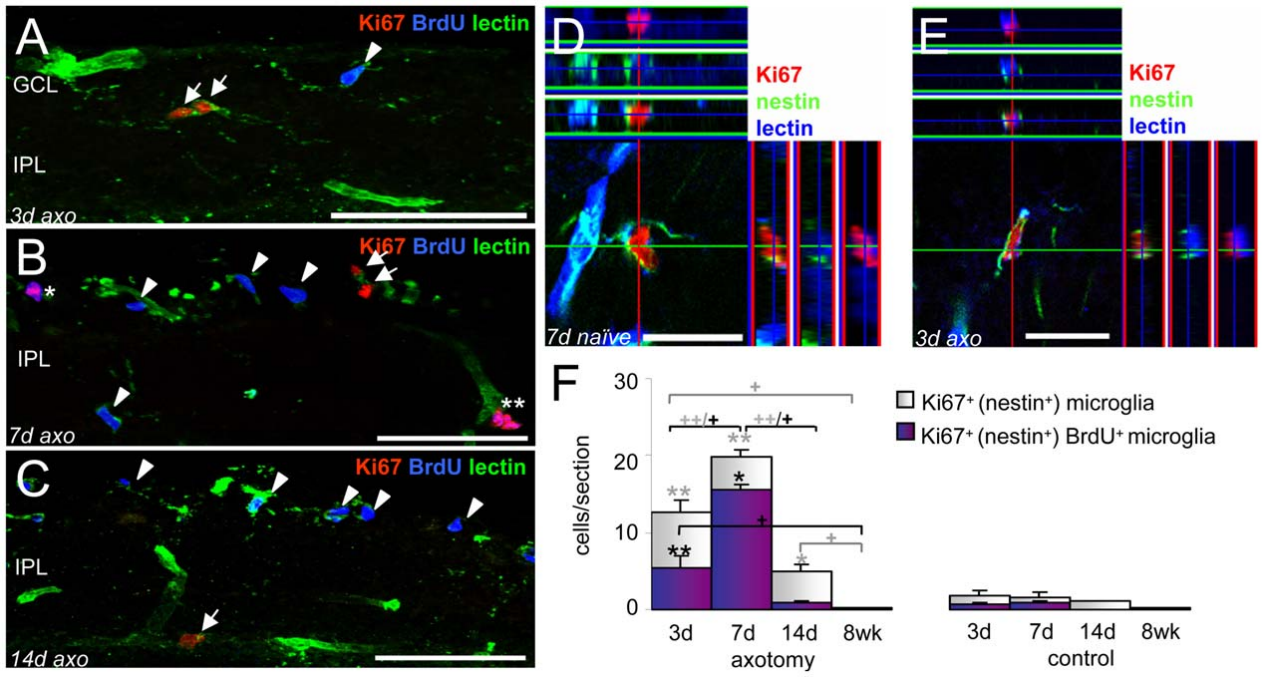
Retinal nestin^+^ microglia proliferated *in situ* in response to ON axotomy. A–E: Immunofluorescent labeling with Ki67 (red), tomato lectin (A–C green; D,E blue), BrdU (A–C blue), and nestin (D,E green). A–C: within the first 2 weeks, significant numbers of Ki67^+^ microglia (arrows) were observed in the inner retinal layers, and Ki67 was partly co-localized with BrdU (arrowheads, co-localization is shown by asterisks). In unlesioned (D) and lesioned tissue (E), Ki67 was exclusively found in nestin^+^ microglia which were mainly located in the IPL. F: Absolute numbers of Ki67^+^ and Ki67^+^BrdU^+^ microglia 3, 7, 14 days, and 8 weeks after ON transection and in the naïve retina. Numbers of Ki67^+^ microglia were increased 3–14 days after ON axotomy compared to corresponding controls, reaching a maximum after 7 days. Maximum of Ki67^+^BrdU^+^ cells was also found 7 days after lesion. Mean ± S.E.M., significant differences between lesion and corresponding control groups (* p<0.05, ** p<0.01), and between the lesion or control groups over time (+ p<0.05, ++ p<0.01) are indicated: grey and black symbols are used for the white and purple diagrams, respectively. GCL: ganglion cell layer, IPL: inner plexiform layer, axo: axotomy. Scale bar in (A–C) 50 µm, (D,E) 20 µm.

Cumulative BrdU labeling allowed for evaluation of additive cell proliferation over time. Therefore, Ki67 labeling was additionally used to evaluate *in situ* proliferation of retinal microglia at the four time points to further support the interpretation of the time course of cell division after ON lesion. In Figs. 6A–C, BrdU^+^ (arrowheads), Ki67^+^ (arrows) and BrdU^+^Ki67^+^ microglia (asterisks) are shown in the acute phase after ON transection. Unexpectedly, all *in situ* proliferating Ki67^+^ microglia co-expressed nestin in naïve controls (Fig. 6D) as well as after ON axotomy (Fig. 6E). Contrary to the previous interpretation regarding BrdU^+^ fractions, this finding suggests that the fraction of BrdU^+^nestin^−^ microglia represent the progeny of the nestin^+^ population that down-regulated or degraded nestin filaments after mitosis, and does not signify a delayed proliferating microglial population. Moreover, microglial nestin filaments appear to account for local microglial proliferation, in particular after ON axotomy. The number of Ki67^+^ (nestin^+^) microglia 3 days after ON lesion was 6 fold higher than in corresponding controls (Fig. 6F). The highest number of *in situ* dividing microglia was found 7 days after axotomy. After 14 days, only 5 Ki67^+^ microglia per section were seen and after 8 weeks, *in situ* proliferation was sparsely observed (one cell per 3–4 analysed sections). Very few Ki67^+^ cells were found in the naïve retina (1–2 cells/sections) independent of the analyzed time point, indicating a physiological microglial renewal.

The number of Ki67^+^ (nestin^+^)BrdU^+^ microglia representing true *in situ* proliferating cells at the different time points are shown in Fig. 6F. Three days after ON axotomy, about 6 BrdU^+^Ki67^+^ cells/section were found (controls: 1 cell/section), which represents approximately 17% of all BrdU^+^ microglia, signifying that 83% BrdU^+^Ki67^−^ had previously divided. In addition, 3 days after ON axotomy, roughly 45% of all Ki67^+^ cells were BrdU^+^. Thus, the majority of *in situ* proliferating microglia were BrdU^−^ (55%), indicating a short time window for BrdU labeling. Hence, most of the cells reside in one of the remaining 3 phases of the cell cycle and do not (yet) acquire the BrdU label during the S-phase. However, although the maximum number of BrdU^+^Ki67^+^ microglia were found 7 days after axotomy (representing approx. 80% of all *in situ* proliferating microglia) this fraction only represents about 10% of all BrdU^+^ microglia estimated at this time point. Thus, 90% of all BrdU^+^ microglia found 7 days after ON axotomy had previously divided. No relevant *in situ* proliferation was found at 14 days and at 8 weeks following axotomy. We conclude that in the adult rat retina, microglial cell division predominantly occurred within one week after surgery.

### Microglial phenotypes in the naïve and lesioned adult rat retina

**Figure 7:**
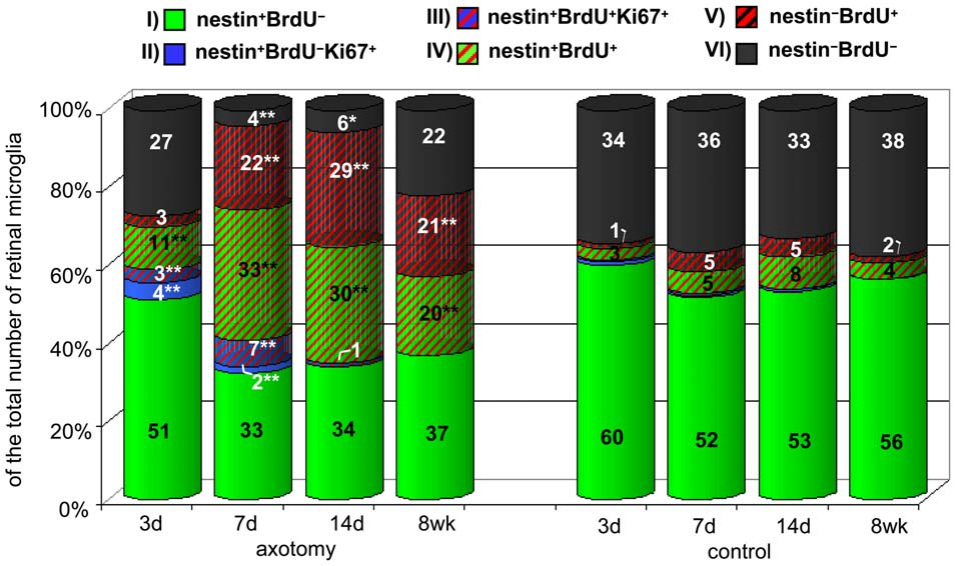
Percentages of the six determined microglial phenotypes after ON transection and in the naïve retina. The nestin^+^ fractions are indicated by a green and nestin^−^ fractions by a dark background. The fractions of BrdU^+^ microglia are illustrated as red-shaded columns. The *in situ* proliferating Ki67^+^ (always nestin^+^) fractions are shown as blue columns. The six resulting phenotypes were defined as follows: I) non-proliferative nestin^+^ microglia; II) *in situ* proliferating nestin^+^ microglia not in the S-phase of the cell cycle (BrdU^−^); III) *in situ* proliferating nestin^+^BrdU^+^; IV) nestin^+^BrdU^+^ microglia previously labeled in the S-phase (had already left the cell cycle); V) nestin^−^BrdU^+^ microglia which were degraded or had replaced their nestin filaments after cell division, and, VI) nestin^−^BrdU^−^ microglia. Significant differences for a particular phenotype between the lesion and control group at a specific time point are indicated by * p<0.05, ** p<0.01.

In the present study, six microglial phenotypes in varying proportions were determined after ON axotomy over the 4 different time points analyzed (Fig. 7). *Phenotype I* represents the nestin^+^ non-proliferating (BrdU^−^, Ki67^−^) microglia capable of proliferating *in situ* and are the responding population after ON axotomy, resulting in *phenotype II* (nestin^+^Ki67^+^ microglia) which is BrdU^−^ and has not yet passed the S-phase. Microglia passing the S-phase are classified as *phenotype III* (nestin^+^Ki67^+^BrdU^+^ microglia). Both phenotype II and III were only found at notable levels on days 3 and 7 after injury, indicating that local microglial division occurs predominantly within one week after ON axotomy. Nevertheless, Ki67^+^ microglia were also observed 8 weeks after lesion with cell numbers similar to naïve controls (for both fractions representing less than 1% of total microglia; 1–2 cells/sections, Figs. 6F,7) indicating physiological self renewal. On leaving the cell cycle (Ki67^−^), microglia acquire the *phenotype IV* (nestin^+^BrdU^+^ microglia). As early as 3 days after ON axotomy, phenotype IV was significantly increased compared to the corresponding control. After 7 days, there was a three-fold increase compared to the fraction obtained at 3 days (p = 0.004) that remained unchanged till day 14. After 8 weeks, 20% of the total microglia consisted of BrdU^+^nestin^+^. This fraction was significantly higher than in corresponding controls and in comparison to the fraction obtained after 3 days, denoting a long-lasting phenotype. Interestingly, this persisting microglial nestin expression is not only associated with cell proliferation as evidenced by Ki67 expression in a few cells even after 8 weeks. Hence, it appears that microglial nestin expression is also required for different cellular processes. *Phenotype V* constitutes microglia that have divided (BrdU^+^) and either degraded, or replaced their nestin. Following ON axotomy, the fraction of phenotype V at day 3 was not different from controls, however, there was a significant increase after 7 days which then remained unchanged over time. In fact, even 8 weeks after ON lesion, phenotype V was significantly higher than the fraction found in controls specifying a long-lasting phenotype. Moreover, in naïve controls as well as 14 days and 8 weeks after lesion, phenotype IV and V seemed to be in equilibrium, indicating an intrinsic mechanism of regulation. Finally, *phenotype VI* comprises undivided, nestin^−^ microglia.

### Nestin^+^ microglia co-express NG2 and vimentin but not GFAP

**Figure 8:**
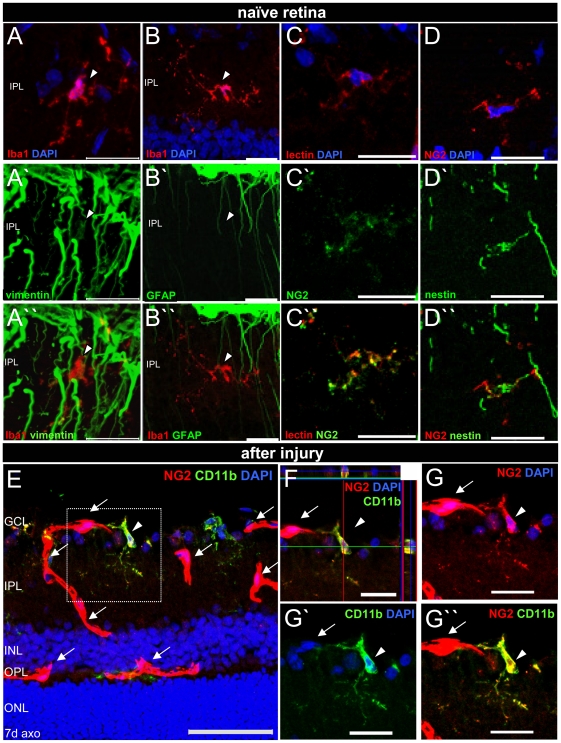
Retinal microglia express vimentin and NG2 but not GFAP. Immunofluorescent labeling with Iba1 (A,B, A″,B″ red), vimentin (A′,A″ green), GFAP (B′,B″ green), fluorescein labeled tomato lectin (C,C″ red), NG2 (C′,C″green, D,D″, E-G,G″red), nestin (D′,D″ green), CD11b (E,F,G′,G″green), and DAPI (A-G, blue) on retinal slices. A–D: ramified retinal microglia contained vimentin filaments in their processes and within the soma, but no GFAP. Resting retinal microglia also expressed NG2 on their surface and were nestin^+^. E-G″: after injury, NG2^+^CD11b^+^ microglia (arrowhead), that can be clearly distinguished from NG2^+^CD11b^−^ pericytes (arrows) displayed an increased NG2 immunoreactivity on their surface. The micrographs in A–E,G-G″ are merged z-stacked images of 1 µm optical sections to illustrate the entire cell dimension. GCL: ganglion cell layer, IPL: inner plexiform layer, INL: inner nuclear layer, OPL: outer plexiform layer, ONL: outer nuclear layer, axo: axotomy. Scale bar in (A-D″,F′-G″) 20 µm, (E) 50 µm.

Ramified Iba1^+^ microglia in the retinal parenchyma also expressed the intermediate filament protein vimentin in some processes (Figs. 8A-A″), whilst GFAP was not determined (Figs. 8B-B″, Table 2). Resting retinal microglia in naïve tissue displaying a weak immunoreactivity for the chondroitin sulfate proteoglycan NG2 was indeed a novel finding here (Figs. 8C-C″). Moreover, parenchymal NG2^+^ microglia belong to the nestin^+^ population of retinal microglia (Figs. 8D-D″), and no NG2^+^nestin^−^ microglia were observed in this study.

**Table 2:** Marker expression in retinal glia and blood vessel cells of naïve and lesioned tissue.

*MARKER*	*MICROGLIA*		*ASTROCYTES*		*MÜLLER GLIA*		*PERICYTES*		*ENDOTHELIAL CELLS*	
	naïve	lesion	naïve	lesion	naïve	lesion	naïve	lesion	naïve	lesion
**nestin**	+	+++	+	+++	+	+++	++	++	++	++
**vimentin**	+	+++	+	+++	+	+++	x	x	x	x
**NG2**	+	+++	−	−	−	−	++	++	−	−
**GFAP**	−	−	+	+++	+	+++	−	−	−	−
**glutamine synthetase**	−	−	+	+	+	+	−	−	−	−
**Iba1**	+	+++	−	−	−	−	−	−	−	−
**CD11b**	+	+++	−	−	−	−	−	−	−	−
**tomato lectin**	+	+++	−	−	−	−	−	−	++	++
**BrdU**	+	+++	−	+	−	−	−	−	−	−
**Ki67**	+	++	−	−	−	−	−	−	−	−

(+): positive cells; (+++): increase in cell number; (−): not found; ( ): increase in immunoreactivity; (x) not analysed.

Three days after ON axotomy, retinal microglia, found mainly in the inner plexiform layer (IPL), displayed weak NG2 immunoreactivity with a dotted labeling pattern similar to the one seen for microglia in unlesioned retinae. Seven days after injury, NG2^+^CD11b^+^ microglia (Fig. 8E, the arrowhead-marked cell is shown in F,G-G″) that were clearly distinguishable from NG2^+^CD11b^−^ pericytes (Fig. 8E-G″, arrows, Table 2) displayed a stronger NG2 immunoreactivity compared to unlesioned tissue. This process was accompanied with an increase in the cell number, especially in the IPL and the GCL. A number of NG2^+^ microglia were BrdU^+^, but all co-expressed nestin. However, in activated microglia, no increase in immunoreactivity for vimentin was apparent. In addition, after ON axotomy, no microglial GFAP expression was found. GFAP was restricted to glutamine synthetase^+^ Müller glia and astrocytes that also express nestin and vimentin, but no microglial markers (Table 2). Finally, within 8 weeks after injury, we did not detect neuronal markers such as Doublecortin (Dcx), TUJ1, NeuN, or Brn3a in BrdU^+^ microglia suggesting a transdifferentiation of this retinal microglial population.

### Nestin^+^NG2^+^ microglia phagocytose apoptotic RGCs

**Figure 9:**
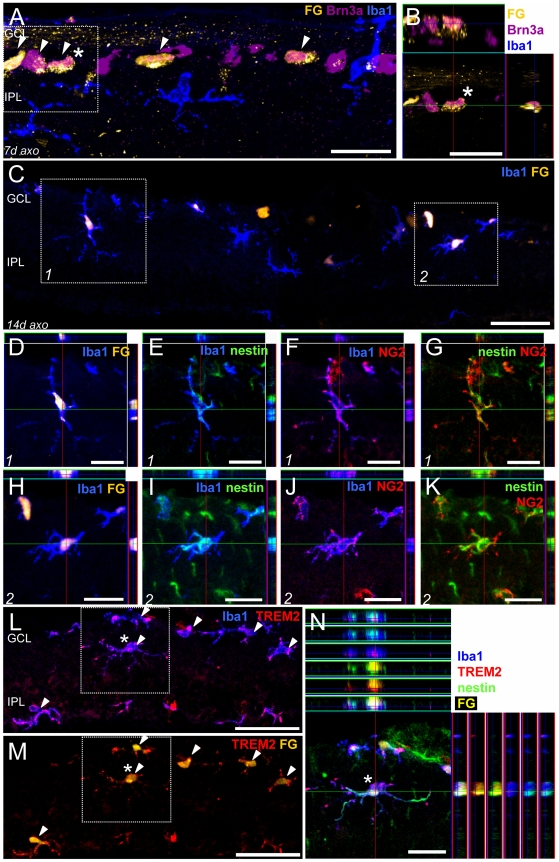
Nestin^+^ and NG2^+^ microglia phagocytose RGCs. Immunofluorescent labeling with Fluorogold dye (FG, A–D,H,L–N gold) as well as Brn3a (A,B magenta), Iba1 (A–F,H–J,L,N blue), nestin (E,G,I,K,N green), NG2 (F,G,J,K red), and TREM2 antisera (L–N red). 7 days after ON lesion, most of the Brn3a^+^ RGCs are retrogradely labeled with FG (A, arrowheads; the asterisk-marked cell in the box is shown in B as ortho view). 14 days after injury, retinal Iba1^+^ microglia have phagocytosed FG^+^ retinal ganglion cells and have incorporated the golden dye (C, box 1 is shown in higher magnification D–G, box 2 in H–K). Phagocytosing microglia were nestin^+^ (E,I) as well NG2^+^ (F,J), merged in (G,K) and expressed the TREM2 receptor on their surface (L,M, the asterisk marked cell in L,M is shown as ortho view in N). Every FG^+^ microglial cell was also TREM2^+^ (arrowheads).The micrographs in A,C,L,M are merged z-stacked images of 1 µm optical sections to illustrate entire cell dimension. GCL: ganglion cell layer, IPL: inner plexiform layer, axo: axotomy. Scale bar in (A,B,N) 20 µm, (C,L,M) 50 µm, (D–K) 10 µm.

Transected Brn3a^+^ RGCs of the GCL can be retrogradely labeled by the fluorescent dye Fluorogold (FG) (Fig. 9A arrowheads, the asterisk-marked cell in the box is shown in B). Fourteen days after ON transection, FG was incorporated by Iba1^+^ microglia that had phagocytosed FG^+^ apoptotic RGCs (Fig. 9C, box 1 and 2 are shown in D–G and H–K, respectively, Video S3). Interestingly, these phagocytosing microglia displayed a ramified morphology (Figs. 9D,H) and expressed nestin (Figs. 9E,I) and NG2 (Figs. 9F,J, merged in G,K). Phagocytosing microglia also expressed the TREM2 receptor (Figs. 9L,M, the asterisk marked cell in the box is shown as ortho view in N) that is responsible for binding and uptake of apoptotic neurons on their surface. All TREM2 microglia were FG^+^ and *vice versa* (Figs. 9L,M arrowheads). This suggests that both nestin and NG2 are not only associated with mitosis, but also seem to play a role in morphological changes associated with migration (move to RGCs in the GCL) and phagocytosis.

### Isolation of retinal microglia expressing nestin, NG2 and vimentin by immunopanning

**Figure 10:**
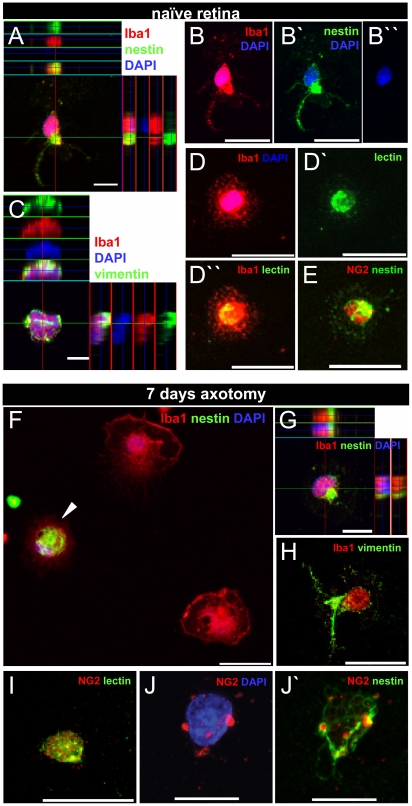
Isolated retinal microglia express nestin, vimentin and NG2. Immunofluorescent labeling with Iba1 (A–D,D″,F-H red), nestin (A,B′,E–G,J′ green), vimentin (C,H green), tomato lectin (D′,D″,I green), NG2 (E,I,J,J′ red), and DAPI (A–D,F,G,J blue) on isolated cells after immunopanning. A–E: isolated Iba1^+^ microglia of naïve retinas co-expressed the intermediate filaments nestin (A,B-B″) and vimentin (C), that were observed in the processes (B,B′) as well as around the nucleus (A,C). Isolated microglia were co-labeled with tomato lectin, further confirming the microglia identity (D-D″). Immunopanning also corroborated that some of the resting retinal microglia were NG2^+^ and that these cells belonged to the nestin expressing microglial fraction (E). F-J′: 7 days after ON axotomy some Iba1^+^ microglia were nestin^+^ (F, arrowhead). Nestin (F,G) and vimentin filaments (H) were found around the nucleus as well as in some processes of Iba1^+^ retinal microglia. In addition, isolated microglia expressed NG2 after injury (I,J,J′) and these cells were also nestin^+^ (J′). The micrographs in B-B″,D–F,H,I are merged z-stacked images of 1 µm optical sections to illustrate entire cell dimension. In J-J′, a single micrograph from the middle of the z-stack is shown in higher magnification. Scale bar in (A,C,G) 10 µm, (B-B′,D-D″,E,F,H-J′) 20 µm.

Microglia isolation by immunopanning was used to confirm specific nestin, NG2, and vimentin expression (Fig. 10). Isolated Iba1^+^ retinal microglia specifically expressed the intermediate filament proteins nestin (Figs. 10A-B″) and vimentin (Fig. 10C). Both filament proteins were arranged in compact filament bundles around the microglial nuclei. Moreover, isolated round microglia of naïve retinae immunolabeled for Iba1 and tomato lectin (Fig. 10D-D″) displayed a strong immunoreactivity for the chondroitin sulfate proteoglycan NG2 (Fig. 10E), possibly due to the reduced cell surface leading to a high density of the surface molecule and, therefore, to a more intense labeling compared to that in the retinal sections. Thus, immunopanning confirmed that NG2 is expressed in resting retinal microglia and that NG2^+^ microglia indeed belong to the nestin^+^ subset of retinal microglia (every scanned cell was positive for both markers).

Iba1^+^ retinal microglia isolated by immunopanning having maximum cell numbers 7 days after ON axotomy also expressed nestin (Fig. 10F, arrowhead) mainly around the nucleus (Fig. 10G). Moreover, the ratio of nestin^+^ microglia to the total number of Iba1^+^ microglia was similar to the numbers of microglia found in the retinal sections (approx. 75%, controls: approx. 60%). After ON axotomy, Iba1^+^ microglia expressed vimentin (Fig. 10H) and displayed a similar pattern as already observed for microglia isolated from naïve retinae. Moreover, NG2^+^ tomato lectin^+^ microglia (Figs. 10I,J,J′) co-expressed nestin (Fig. 10J″). Furthermore, microglial GFAP expression or that of neuronal markers was not found in isolated cells. Finally, labeling specificity of isolated microglia was confirmed by using negative controls without primary antibodies (not shown).

Thus, in the adult rat retina, a subpopulation of microglia express nestin, NG2, and vimentin.

## Discussion

### The majority of resting retinal microglia express nestin

In the present study, the majority of parenchymal microglia in the naïve adult rat retina expressed nestin. Interestingly, retinal microglia displayed a similar subcellular distribution of nestin filaments as reported in a recent study in the brain [29]. However, the fraction of resting retinal microglia expressing nestin (60%) that we found was higher than previously reported for various brain regions e.g., the cerebral cortex (24%), or the dentate gyrus (38%), probably because the retina differs from other brain regions in its function as a sensory organ.

Nestin, an intermediate filament, serves as a marker of proliferating and migrating precursor cells in various tissues, including muscle, testis, skin, kidney, vasculature, and the developing CNS (see reviews [51], [52]). Nestin, in the CNS including the neural retina, is down-regulated upon differentiation [53], [54] and replaced by other intermediate filaments, i.e. GFAP in glia and neurofilament/α-internexin in neurons [51]. Notably, in the present study, nestin^+^Iba1^+^ cells were also found in the ciliary epithelium, which, like the neural retina, is also of neuroectodermal origin [53], [55]. However, in the choroid or the ciliary stroma, both tissues of mesodermal origin, all Iba1^+^ cells, described as macrophages [56], [57] were nestin^−^. In addition, circulating monocytes have also been shown to be nestin^−^ [58] suggesting an environmentally-dependent effect on microglial nestin expression.

We additionally viewed horizontally oriented nestin^+^ structures within the retina that were identified as blood vessel cells, an observation in accordance with other reports [26], [50], [59]. However, a recent study showed that nestin is only expressed in proliferating endothelial cells, and not in the mature vasculature, further indicating that nestin^+^ cells constitute rather immature cells [60].

Interestingly, in the naïve retina, we observed a few microglia dividing *in situ* which is consistent with our previous work in mice [10]. Notably, all of the cells described in the present study were nestin^+^. Thus, we conclude that microglial nestin expression may play a role in microglial proliferation pointing to a physiological self-renewal of the population.

### Nestin^+^ microglia are the responding in situ proliferating population after ON transection

After ON axotomy, retinal microglia increased, and cell numbers as well as distribution patterns were consistent with previous reports in rats [61], [62]. The increase was primarily due to local microglial cell division as shown by Ki67 labeling and was in accordance with our previous report in mice showing that distal ON transection, which does not affect integrity of the BRB, is an appropriate model for analysis of the proliferative potential of local microglia [10]. Herein, we show for the first time that ON transection leads to an expansion of the nestin^+^ microglial subpopulation reaching maximum cell numbers within 2 weeks after injury. In contrast to brain microglia [16], [17], nestin expression was not only observed in ameboid, but also in ramified microglia. However, the most interesting finding was that every single Ki67^+^ *in situ* dividing cell was nestin^+^ and led us to the conclusion that only nestin^+^ microglia divide in response to injury and, therefore, every BrdU^+^ microglia was nestin^+^ at cell division. Thus, nestin^−^BrdU^+^ microglia are progeny of nestin^+^ microglia and not an independent subpopulation that may proliferate later, as we previously assumed. Microglial nestin expression in association with cell cycle re-entry has not yet been reported, although nestin expression is correlated with proliferating NPCs [32], [33], [63], cultured neurogenic astrocytes [64], reactive astrocytes [25], [65], ependymal cells in the spinal cord [66], mesangial cells of the kidney [30], and intestinal [31] and brain [60] epithelial cells. A recent study demonstrated that reduction in nestin expression resulted in a G1 cell cycle arrest as well as in a lowering of cortical neurogenesis [63]. Furthermore, blocking nestin expression by using nestin-morpholino showed nestin as being essential for brain and eye development in Zebrafish since loss of nestin lead to apoptosis of NSC/PCs [67]. Interestingly, nestin is thought to be responsible for NSC/PC proliferation via promoting the activation of PI3K in response to mitogenic growth factors [63]. However, nestin expression did not exclusively correlate with in situ proliferation because 8 weeks after axotomy there was still an increased fraction of nestin^+^BrdU^+^ microglia which was higher than that obtained 3 days after injury. Since there was no detectable *in situ* proliferation after 2 and 8 weeks, we suggest that two different phenotypes persist for several weeks after injury, one with transient cell cycle- dependent, and one with prolonged cell cycle-independent nestin expression, which both arise from a common nestin^+^ phenotype. Moreover, nestin^+^ and nestin^−^ phenotypes appear to maintain a physiological equilibrium. This equilibrium is re-established several weeks after ON injury signifying an intrinsic mechanism of regulation.

Nestin^+^ microglia additionally co-expressed the intermediate filament protein vimentin, a further component of the cytoskeleton. Vimentin has been observed in brain microglia after facial nerve axotomy [47]. More recently, vimentin was also found overlapping with nestin expression in resting brain microglia and appears to maintain structural integrity and cell shape [29]. However, vimentin is also expressed in undifferentiated/immature neural cells [68], [69], [70], [71]. This is not surprising since vimentin is co-expressed and acts in concert with nestin [33], [72]. Nestin is unable to polymerize by itself, but rather constitutes heterodimers with vimentin [73] allowing for and retaining the flexibility of the intermediate filament network that is a requirement for cell proliferation and migration of, e.g. neural progenitor cells (NPCs) [32], [73]. Hence, we conclude that both intermediate filaments, nestin as well as vimentin are required for microglial cell proliferation and migration and are consequently expressed in numerous microglia during acute phase after ON lesion. GFAP was not expressed in resting or activated microglia, a fact consistent with previous studies in developing [24], adult naïve [29], or lesioned adult brain [21]. In addition, this nestin^+^vimentin^+^ subset of retinal cells proliferating *in situ* in response to ON axotomy was also negativ for glutamine sythetase, and therefore, does not belong to the macroglial cell lineage.

### Microglial nestin-NG2 co-expression in the naïve rat retina

Here, we show for the first time that resting nestin^+^ microglia of the naïve retina displayed weak NG2 expression, an observation not yet made for other brain regions [18], [74]. This novel finding was further confirmed by the labeling of isolated retinal microglia after immunopanning and may suggest heterogeneity of retinal and cerebral microglia. NG2 is a marker for oligodendrocyte precursor cells (OPCs) [22], [75] belonging to a glial cell class referred to as polydendrocytes [76] or synantocytes [77]. However, polydendrocytes are found only in the optic nerve [78], [79] and not within the retina [80], [81], [82], and, further, do not express nestin [19]. In summary, NG2 glia are morphologically, antigenically, and functionally distinct from microglia [76], [83]. NG2 expression in the naïve adult rat retina has been reported in mural blood vessel cells, i.e. smooth muscle cells and pericytes [84], [85], [86]. The latter display distinctive NG2 labeling predominantly restricted to the soma [86], co-express nestin [26], but no microglial markers, and are also morphologically distinct from parenchymal microglia, as shown herein.

### Increased number of NG2^+^ microglia in the lesioned rat retina

ON transection induced up-regulation of microglial NG2 immunoreactivity as already reported for the brain [19], [21], [87] and spinal cord [18], [20]. In the brain, a number of activated microglia that up-regulate NG2^+^ after lesion were also nestin^+^ [18], [20]. However, there are controversial opinions regarding the origin of these transient NG2^+^ immunological cells. After lipopolysacccharide (LPS) stimulation or neurotoxic injury, there resulted a blood brain barrier (BBB) breakdown, and NG2 was observed on the invading blood-borne cells [74], [88]. Moreover, the transiently induced NG2 expression on these microglia appears to have a role in inflammatory function, in particular, in iNOS induction and cytokine expression [88]. In contrast, after facial nerve axotomy, the BBB is preserved, and NG2^+^ cells arise from endogenous resident microglia through cell proliferation [18], in accordance with our results after ON transection. However, we can not completely exclude the possibility that few invading blood cells also express NG2. Consequently, the appearance and cell number of the different NG2^+^ immunologic phenotypes may be dependent on lesion type and severity. However, as for nestin expression, NG2 expression is restricted to immunological cells that have either entered or reside in the CNS, since blood monocytes are NG2^−^ [20], [87]. Thus, NG2 expression appears to be induced by environmental cues.

We provide evidence that these NG2^+^nestin^+^ microglia already reside in naïve tissue and increase through local cell division after injury. Other studies have recently reported that NG2 plays an important role in cell division and migration processes, particularly for NSC/PCs [83], [89], [90], but also for endothelial cells [91], [92]. NG2 is a transmembrane protein that functions in cell signalling via growth factor or receptor binding and is responsible for the immature cell state of NG2 glia [23]. Interestingly, brain Iba1^+^NG2^+^ cells, also termed BINCs, express a 300 kDa NG2, while polydendrocytes express a post-translationally modified form constituting 290 kDa. Therefore, as already revealed for nestin, microglial NG2 differs from that of neural cells.

### Nestin^+^NG2^+^ microglia phagocytose apoptotic neurons

Using retrograde labeling for retinal neurons, we demonstrate herein that phagocytosing microglia that have incorporated the fluorescent dye were nestin^+^ and NG2^+^ and, moreover, expressed TREM2, a receptor expressed on phagocytosing microglia/macrophages [43], [44]. This suggests that both nestin and NG2 seem to effect changes in cellular shape responsible for phagocytosis and possibly for motility. Two previous brain study reported that NG2^+^ microglia phagocytose neuronal debris, but there no direct evidence for this observation was demonstrated [87], [88].

### Are microglia immature progenitors?

Recently, microglia were suggested to represent a separate population different from specialized macrophages of the CNS [93] and to possess a more immature state [21], [94], [95]. Microglia of the adult CNS appear to be progeny of the neonate microglial population that have to divide for self-renewal. In support of this hypothesis, the adult subpopulation of retinal microglia in the present study displayed evidence for physiological self-renewal, as indicated by BrdU incorporation and Ki67 expression found in naïve tissue. Interestingly, several studies found expression of neural markers on activated microglial cell populations [96], [97] that give rise to neurons *in vitro* [21], [24]. Moreover, these microglia-derived neurons are functional and can generate action potentials [98]. Since there is increasing evidence that CNS injury can induce neural progenitor characteristics in activated microglia of non-neurogenic regions *in vivo* [21], [97], [99], it is conceivable that this retinal microglial subpopulation may represent an “intermediate” cell type, already functional, but not completely committed and, therefore, inducible for transdifferentiation, which may act as an endogenous neural progenitor-like cell after a lesion. In the current *in vivo* study, we were not able to detect neuronal markers in microglia cells indicating transdifferentiation of these cells as reported under culture conditions. Hence, further research is required to elucidate this aspect.

Taken together, we demonstrate that more than 50% of all retinal resting microglia in the naïve adult rat retina express nestin, representing a greater fraction than reported for any other region of the brain. After ON axotomy, the number of these cells increased due mainly to *in situ* cell proliferation, reaching maximum numbers 7 days after injury. The most important finding, however, is that all *in situ* dividing microglia were nestin^+^, indicating that nestin expression is correlated with cell cycle re-entry. Moreover, these findings support the notion that nestin^−^BrdU^+^ cells arise from the nestin^+^ phenotype. We also demonstrate that resting and activated retinal microglia co-express two further neural proteins, vimentin, and NG2. Though the present study revealed that in particular the expression of nestin appears to be affected by the surrounding environment, it is nonetheless required for local microglial cell division and migration after distal ON transection. Further, we revealed that nestin^+^NG2^+^ microglia phagocytose apoptotic RGCs in the acute phase after ON axotomy indicating that nestin and NG2 play a role in changing cell shape and affect cell motility. In addition, we showed that following ON injury, this endogenous population transiently increases in number and is associated with a clean-up function. Finally, we did not observe any transdifferentiation of these cells toward neuronal phenotypes over the 8 week study period. In conclusion, the ON lesion alone is not sufficient to induce the putative multipotent progenitor features of retinal microglia *in vivo*.

## Supporting Information

Video S1 **Nestin filaments in resting microglia.**
(AVI)

Video S2 **Nestin filaments in the cytoskeleton of activated microglia.**
(AVI)

Video S3 **NG2^+^ microglia have phagocytosed FG^+^ RGCs.**
(AVI)
